# Experiences of palliative care for patients with pulmonary fibrosis: A qualitative exploration of patients and partners perspectives

**DOI:** 10.1371/journal.pone.0360649

**Published:** 2026-10-13

**Authors:** Lian Trapman, Lea M. Dijksman, Jan C. Grutters, Saskia C. C. M. Teunissen, Everlien de Graaf

**Affiliations:** 1 Interstitial lung diseases Center of Excellence, member of European Reference Network-Lung, St Antonius Hospital, Nieuwegein, the Netherlands; 2 Department of Value-Based Healthcare, St Antonius Hospital, Nieuwegein, The Netherlands; 3 Division of Heart and Lungs, University Medical Center Utrecht, Utrecht, The Netherlands; 4 Center of Expertise Palliative Care Utrecht, Julius Center for Healthcare Sciences and Primary Care, Department of General Practice, University Medical Center Utrecht, Utrecht, The Netherlands; Kurume University School of Medicine: Kurume Daigaku Igakubu Daigakuin Igaku Kenkyuka, JAPAN

## Abstract

**Background:**

Pulmonary fibrosis is often a progressive disease associated with reduced life expectancy and quality of life. Palliative care aims to optimize quality of life for both patients and their informal caregivers. So far, multiple studies have shown unmet palliative care needs in patients with pulmonary fibrosis. This study explores the experiences and needs of patients with pulmonary fibrosis and their partners to identify opportunities for improving palliative care.

**Methods:**

A qualitative study was performed with focus groups and individual semi-structured interviews with patients and their informal caregivers. Data collection and analysis were performed iteratively. A reflexive thematic analysis was performed, following the methods of Braun and Clarke.

**Results:**

In total, fifteen patients and 10 informal caregivers participated. One focus group, 13 individual interviews, and 5 dyadic interviews were performed. Two themes were generated: ‘See me as a person’, and ‘Guide me through the trajectory’ with both two subthemes, reflecting the patient and partner perspectives. Patients seek recognition beyond their disease, valuing empathy, open communication, and holistic care. At the same time, both patients and partners express the need for clear guidance and support throughout the medical journey, from diagnosis to end of life care, including emotional, practical, and logistical aspects.

**Conclusions/discussion:**

Patients and partners experience a ‘one-size-fits-all’ approach to care and reveal several gaps that can be improved. Patients expressed the importance of being acknowledged as a person beyond the disease and being supported in navigating care and anticipating future needs. Partners highlighted the need to be valued and to receive information that enables them to provide care and maintain a sense of control and security.

## Introduction

Pulmonary fibrosis refers to a group of rare lung conditions characterized by scarring of the lung tissue. Idiopathic pulmonary fibrosis is one of the most common types of Pulmonary fibrosis and is progressive in nature. Other types of progressive fibrosis are fibrotic hypersensitivity pneumonitis and fibrotic ILD related to CTD. Although latter types of pulmonary fibrosis can sometimes be stabilized using immunosuppressant regimes, Idiopathic pulmonary fibrosis and many other progressive Pulmonary fibrosis are relentlessly progressive. While anti-fibrotic drugs can slow progression, most patients face a substantially reduced life expectancy [1–3].

The burden of the disease is usually high for both patients and their partners. Patients often experience dyspnea, coughing, depression, and anxiety, with increasing levels over time as the disease progresses [4,5]. This results in a significantly decreased quality of life [6–8]. To address these, the European Respiratory Society published a guideline in 2023, emphasizing the importance of integration of palliative care into the management of interstitial lung disease [9].

Given the severity of symptom burden and limited life expectancy, patients suffering from progressive pulmonary fibrosis can benefit from palliative care. According to the WHO definition, palliative care is appropriate for patients and their families facing challenges associated with life-limiting illness. It aims to optimize quality of life by alleviating suffering through early assessment and treatment of multidimensional needs [10]. Palliative care consists of multiple elements, such as the management of multidimensional symptoms, addressing concerns and needs, and advance care planning – preparing for the future by early conversations about values, wishes, and needs [11]. This care is delivered by a multi-professional team that emphasizes patient autonomy, access to information, and the right to choose among available options [12]. Specific studies in pulmonary fibrosis populations, however, showed that patients and their partners frequently faced unmet palliative care needs [13–15].

Prior research in patients with pulmonary fibrosis suggested that patients wished to discuss their personal values, goals, and preferences regarding end-of-life care, highlighting the need for healthcare professionals to initiate these conversations [7,16]. Studies involving patients with other incurable diseases indicated that these patients highly valued open and honest communication, as well as being recognized as a person beyond the illness. These patients also expected expertise and a proactive approach from healthcare professionals. In addition, partners often expressed an greater need for information than patients and desired to feel valued and supported [17,18].

Improving the quality of care, in this case palliative care, can be approached by first considering patients’ expectations, then their actual experiences, and finally their unmet needs [19]. While expectations provide a benchmark and needs highlight gaps in care, experience offers the most direct insight into what patients actually encounter in clinical practice. Therefore, in this study, we focus on patients’ and their partners’ experiences, as these are crucial for identifying actionable targets to enhance the quality of palliative care.

## Methods

### Qualitative approach and conceptual framework

A generic qualitative study with patients and informal caregivers between 2021 and 2023. This study was grounded in an interpretivist/constructivist paradigm, assuming that individuals construct their own social realities through interaction and experience [20]. Given our research focus, this paradigm enabled us to approach the topic by prioritizing understanding the participants’ perspectives within their specific contexts. Standards for reporting qualitative research guidelines were used [21](available in the supporting information 1).

In this study, we adopted the World Health Organization (WHO) definition of palliative care, which describes palliative care as an approach that improves the quality of life of patients and their families facing life-threatening illness through early identification, assessment, and management of physical, psychosocial, and spiritual needs [10]. In line with this definition, palliative care in pulmonary fibrosis begins at the time of diagnosis and continues throughout the illness trajectory.

### Researcher characteristics and reflexivity

Data collection was performed by two separate interviewers (JH and LT). JH was trained in interviewing on the job, and LT is a novice qualitative researcher working as an academic nurse and specialist palliative care nurse in training. Both interviewers had an established professional relationship with two patient participants.

The multidisciplinary research team was involved in analyzing data and discussing themes. Coding was done by LT and LD (an epidemiologist and a novice qualitative researcher). An experienced qualitative researcher, EdG (nursing scientist and epidemiologist), supervised this data analysis, and all analyses were discussed within the analysis team. The identified categories and themes were then discussed with the broader research team, including a professor in ILD and a professor in palliative care.

### Context

This study was performed in a referral center for interstitial lung diseases. Patients with pulmonary fibrosis visit the outpatient clinic every three to six months for consultations with the pulmonologist, which typically last 15 minutes. Additionally, patients are offered consultations with a nurse when starting with medication or oxygen therapy, if the pulmonologist determines that the patient needs more support, or at the patient‘s request. The length of these consultations varies depending on the type of support needed.

### Participants and sampling strategy

Patients with any form of pulmonary fibrosis, as diagnosed by their pulmonologists and a multidisciplinary team, and their informal caregivers were eligible for this study, regardless of prognosis. A purposive sample of patients and informal caregivers was selected to achieve optimal diversity in age, sex, and phase of illness. To further ensure diversity, female patients and patients on the lung transplantation list were personally contacted by their healthcare professional. To provide a comprehensive perspective, a bereaved relative was also invited to participate, ensuring that all phases of palliative care, including bereavement care, were represented.

Starting June 1^st^, 2021, eligible patients, informal caregivers, and bereaved relatives were informed about this study through two channels:

1) The pulmonologist introduced the study during consultations with all attending persons and gave the link to sign up
2) The Dutch pulmonary fibrosis patient organization promoted the study using their website (www.longfibrose.nl), newsletter, and social media platforms, inviting patients to sign up using the provided link.

Patients and informal caregivers could register online and indicate their preference for an interview or focus group via the sign-up form. All registered individuals were contacted by the researcher (LT) to assess eligibility. Recruitment ended on October 1^st^, 2022.

### Ethical issues

This study was conducted in accordance with the Declaration of Helsinki [22], Good Clinical Practice [23], and the General Data Protection Regulation [24]. Medical research Ethics Committees United determined that this study is not subject to the ‘Medical Research Involving Human Subjects Act (WMO) (MEC-U W21.018). All participants received a standard patient information folder outlining study aims, procedures, confidentiality, and voluntary participation, as required under Dutch ethical regulations. All participants provided written informed consent for the use of their data in this study prior to the interviews.

Participants’ consent and interview records were documented and securely stored in REDCap (www.project-redcap.org) and pseudonymized by assigning each participant a code number during transcription. Only LT and JH had access to recordings, transcripts, and the pseudonymization document, stored on two different hard disks.

### Data collection methods

A combination of focus groups followed by individual interviews was chosen. First, a focus group was performed to gain insight into relevant topics discussed between patients and informal caregivers.These relevant topics were further explored in depth during subsequent individual semi-structured interviews. Patients and their relatives participated only once in this study and could do so independently or as dyads, based on their preferences. Data collection ended when the research team judged that the dataset provided sufficient depth and interpretative insight to address the research question.

Due to COVID-19 regulations, the focus group was held online using Pexip software, www.pexip.com. Furthermore, interviews could be conducted digitally or face-to-face and located at patient’s home or at the outpatient clinic.

Memos were created after the focus groups and interviews, during transcription, and during data analysis. Data collection and analysis alternated, along with memos that guided adjustments to the interview guide by identifying topics needing further focus. During data collection, the research team regularly reflected on the quality of the interviews and identified topics to emphasize in subsequent interviews.

Two interview guides were developed, for focus groups and semi-structured interviews, based on literature and the research team’s experiences (Supporting information 2).

The guide for the focus groups was designed to facilitate open discussion and identify themes important to patients and informal caregivers. The focus groups began with a broad, open-ended question: “How do you define palliative care?” When rapport was established, additional questions were introduced to explore participants’ experiences, along with requests for specific examples. The focus group leader ensured (LT) that every participant had sufficient time and space to share their opinion and stories.

The interview guide for the semi-structured interviews was developed to encourage patients and informal caregivers to share both positive and negative experiences with palliative care and discuss end-of-life topics with healthcare professionals (HCPs). Based on the experiences in the focus groups, the questions in the interview guide were simplified. During the dyadic interviews, there was additional attention for the involvement of both interviewees and the use of both interview guides for patients and informal caregivers.

### Data processing

The focus groups and interviews were audio-recorded, transcribed verbatim, and anonymized by administrative assistants. Transcripts, along with memos, were uploaded and stored in Atlas.ti.23 using a pseudonymization code. From that point on, all data were processed in Atlas.ti. Interview records were stored in REDCap (www.project-redcap.org) under the same pseudonymization code.

### Data analysis

A thematic analysis was performed following the six phases described by Braun and Clarke [25], here shown in *Italics*. Two researchers (LD, LT) first *familiarized* themselves with the data by reading and re-reading the transcripts. *Initial coding* was performed independently and inductively on two transcripts in Atlas.ti (LT and LD), using open and detailed coding while staying close to participants’ wording.

Based on this initial coding, a preliminary coding framework was developed and applied to the subsequent transcripts (LT, LD). Each interview was discussed to reach a shared understanding and refine the interpretation.

Codes were then organized into meaningful clusters, first manually and later using Atlas.ti functions. These clusters formed the basis for developing *potential themes* and subthemes, which were *reviewed*, *refined*, and *named* through discussion with the multi-professional team.

Theme development was approached as an active and interpretative process, consistent with reflexive thematic analysis, rather than as a passive emergence from the data.

### Techniques to enhance trustworthiness

To enhance **credibility**, memos were written after the focus groups and each individual interview, during transcription, and throughout the data analysis process. Member checking was performed by providing short summaries during focus groups and interviews to ensure participants’ information was well understood [26].

To ensure **dependability,** an audit trail was maintained throughout the whole process of data collection and analysis, documenting all decisions made during the study. Coding and theme development were discussed and reviewed by multiple researchers to strengthen consistency.

**Transferability** was supported by providing detailed descriptions of participants’ roles, experiences, and the study’s context, enabling us to assess the applicability of findings to other settings.

**Confirmability** was promoted using reflexive memos and regular team discussions, which helped minimize interviewers’ and researchers’ biases and ensure that interpretations were grounded in the data.

## Results

After interviewing 15 patients and 10 informal caregivers, no new information related to the research question were found. While we tried to recruit a variety of informal caregivers, only partners of patients volunteered to participate.

Only three patients signed up for the focus groups, of which two were able to participate, and the focus group took 60 minutes. Subsequently, 13 individual interviews and 5 dyadic interviews were conducted. The interviews lasted 54 minutes on average (range: 28–129 minutes). Patients were mainly male (87%) and diagnosed with idiopathic pulmonary fibrosis (87%). All informal caregivers were partners of patients, and one was a bereaved partner. All participants’ characteristics are shown in Table 1.

**Table 1:** Participants’ characteristics.

	Patients (n = 15)	Informal caregivers (n = 10)
Male	13 (87)	2 (20)
Age	72.5 [68.5-76.5]	58 [55-69]
Diagnosis		
IPF	13 (87)	
FPF	1 (6)	
Autoimmune related pulmonary fibrosis	1 (6)	
Educational level (ISCED)		
3	0	1 (10)
4	6 (40)	2 (20)
5	3 (20)	1 (10)
6	3 (20)	0
NA	3 (20)	6 (60)
Social roles in daily life		
Retired	14 (94)	9 (90)
Working	1 (6)	1 (10)
Living with partner	14 (94)	9 (90)
Living alone	1 (6)	1 (10)
Location of the interview		
Digital	11 (73)	2 (20)
Face to face at home or at clinic	4 (27)	8 (80)
Focus group	2 (13)	0
Single interviews	8 (53)	5 (50)
Duo interviews	5 (33)	5 (50)
Partner	–	10*

All variables are in n (%), or median [25%−75%]. ISCED = International Standard Classification of Education (ISCED [27]):3: Upper secondary education, 4: Post-secondary non-tertiary education, 5: Short-cycle tertiary education, 6: Bachelor’s or equivalent level. IPF = Idiopathic pulmonary fibrosis, FPF = Familial pulmonary fibrosis. * Of which one was a bereaved partner.

From the data, two main themes were generated: **“See me as a person”** and **“Guide me through the trajectory.”** Within these themes, the perspectives of patients and their partners differ slightly, as reflected in the subthemes.

### See me as a person

Participants expressed the desire to be seen and recognized. Patients wanted to be seen as a person with all the different aspects in life, while partners emphasized feeling valued and included in the care process- needs that were often unmet.

#### See me as a person, with all the different aspects in my life.

Patients experienced their relationship with healthcare professionals in various ways. They consistently appreciate and expect open, honest conversations with compassion and full attention. Most patients perceived continuity of care, seeing the same healthcare professional over time, as comforting and beneficial. However, most consultations focused on test results and treatment follow-up, leaving many patients feeling unseen beyond their disease.

While many value a personal connection, others prioritize expertise. For them, interactions are more transactional, focusing strictly on medical knowledge rather than relationship building. Despite these differences, expectations are not explicitly discussed with their healthcare professional.

*“When you come in [the consultation room], they pull the computer towards them and say: oh, you are relatively stable. Well, and then asking further questions often becomes difficult at such a moment.” (Patient 1)*

Many patients expressed a need for a more multidimensional approach in which healthcare professionals address not only disease and physical consequences, but also psychological and social challenges. Participants noted that the emotional burden of the disease is often overlooked.

*“Emotional, I am often emotional. It happens more and more that I get short of breath. I mean, even when I haven’t done any physical work, but sometimes the emotions themselves can really affect me.” (Patient 5)*

Those with familial pulmonary fibrosis highlighted additional concerns such as work, family history, and potential risks for their children, which they felt were rarely acknowledged or adequately addressed.

“*At the end of the month, I will turn 59. Yes, that is very young. Yes, then I am at a very different stage in life compared to someone who is 70 and receives a diagnosis like this.” (Patient 13)*

Furthermore, participants noted that healthcare professionals often underestimate the emotional impact of information, particularly around diagnosis. Some reported receiving critical updates by phone or searching online for prognosis details, which they found distressing. While some empathized with the difficulty of delivering bad news, most preferred receiving such information in person and sensitively.

#### Recognize and value me and my needs as a partner.

Many partners reported feeling excluded from medical conversations and not recognized as part of the patient’s support system, both as partners and as informal caregivers. Additionally, not all partners were present at the time of diagnosis, which led to a delayed understanding of the severity of the disease.

*“No, I feel completely unseen as a partner. He is the patient. He is the one he [the doctor] talks to, and who he [the doctor] looks at.” (Partner 13)*

Partners often described taking an active role during medical consultations by paying close attention, taking notes, and seeking clarification when needed, to compensate for the patient’s limited recall.

*“Usually, my wife comes along. And sometimes she asks questions as well, and I find that very pleasant, because she has a different perspective. You hear a bit more in that way. Moreover, she listens to the answers, and I tend to forget the answers after the conversation.” (Patient 9)*

Both patients and partners noted that this involvement sometimes adds complexity, as they sometimes withhold or filter information to protect each other.

*“But you are together, and you want to protect each other against certain things. And I think he [the patient] protects me a lot too.” (Patient 17)*

### Guide me through the trajectory

*“Guide me through the trajectory”* refers to a need expressed by participants for both information and practical guidance. Patients required support and guidance to be able to bear what happens, but also to anticipate future needs, including navigating the healthcare system. Partners focused on receiving information that enables them to provide optimal informal care while also offering a sense of control and safety.

#### Walk with me through this journey.

*Help me understand what’s ahead, explain what I need to know, and guide me through the healthcare system. More than anything, let me feel I’m not going through this alone.*

Most patients expressed feelings of struggle and loneliness in this trajectory. They experienced a lack of clear, reliable information about the disease, and when information was provided, the quality and content varied among healthcare professionals. Written materials were not always perceived as sufficient. Most patients searched the internet for information, but doubted the reliability, while others avoided searching out of fear. All participants expressed a need for timely information adjusted to their unique circumstances. Preferences varied: some wanted comprehensive details at diagnosis, others preferred a more dosed approach. Regardless of timing, adequate information was considered essential for making appropriate decisions about future treatment and care plans.

*“What I am looking for is information about what to expect, what is possible, what scenarios might occur, what happens within those scenarios, and how I can respond if A happens, B happens, or C happens. I really feel the need to know. ” (Patient 3)*

*“The pulmonologist actually just gave me a booklet. I then reached out to the pulmonary fibrosis association myself, and they provided me some additional information. But that was pretty much it.” (Patient 14)*

Almost all patients wanted more information about their prognosis, not just survival time, but also what to expect as the disease progresses. Some mentioned they refrain from asking questions, not out of hesitation or discomfort, but because they anticipated vague or uncertain answers from healthcare professionals.

*“Because I really see that as a black hole, truly. That doesn’t seem like something for the pulmonologist, because even though they could do it, they are much more focused on things like: what are the test results, what are the facts, and what do those facts say in relation to the past and the future? They might say, ‘Well, maybe it would be wise for you to start doing this or that.’ Of course, they do say that. But that’s all more short-term, the next step, so to speak. But I also need a conversation about the steps beyond that.” (Patient 3)*

Patients felt uncertain and worried when information was lacking or not tailored to their needs. This made it difficult to explain their disease to others and often led to multidimensional problems such as anxiety, work-related problems, and strained social relationships.

Almost all agreed that end-of-life conversations are confronting, yet necessary. Most did not feel comfortable initiating these discussions, were unsure whom to approach, and lacked clarity about how to ask for more information or talk about their future. Despite this hesitation, participants acknowledged that without such conversations, concerns and worries remained unresolved.

*“Of course, that is confronting [talking about end of life]. Of course. But it’s also good to know how the planning will go. If you know from start to the end… Then I know what to expect.” (Partner 19)*

Most participants only mentioned the pulmonologist when discussing healthcare professionals. When asked about their relationship with the nurse, most patients were unaware of the nurse’s role or said they contacted the nurse only for prescription renewals. A few participants had direct contact with a nurse and felt informed and supported by this contact.

*“I speak to the nurse often, every two weeks. They respond very quickly and take my questions seriously. It feels very accessible”. (Patient 14)*

*“In the beginning, shortly after my diagnosis, I felt supported here at the ILD clinic. I also felt somewhat heard- people were genuinely interested in what they could do for me. But now that the contact with the nurse is gone, I no longer experience palliative care.” (Patient 3)*

Some participants mentioned the general practitioner as their primary point of contact for future care and concerns and therefore did not see a larger role for the pulmonologist or nurse. contrary to this, some patients do not see a role for the GP and rely on the pulmonologist, linking this preference to the rarity of the disease, which fosters greater trust in the specialized healthcare professionals.

*“Sketching out future scenarios and addressing the challenges you face, that is something the general practitioner can not do, as they lack that expertise.” (Patient 3)*

#### Give me the information I need to take care of my partner.

*Help us navigate the healthcare landscape, to find care for my partner, and for me.*

Partners described that, as the disease advances and the patients’ physical abilities decline, their role becomes increasingly critical. Many, however, feel unprepared for these responsibilities, often lacking the knowledge and information needed to provide adequate care or respond in acute situations. This uncertainty contributes to heightened anxiety about the future. This uncertainty contributes to heightened anxiety about the future. In addition to hands-on care, partners frequently coordinate medical appointments as the patient’s capacity diminishes, expressing a strong need for guidance in navigating the healthcare system.

*“One day, he will be struggling to breathe. What am I going to do? What should I do?” (Partner 17)*

Beyond practical challenges, partners also seek social and psychological support, commonly experiencing anticipatory grief, and emphasizing the need for time, space, and support to cope with the situation. Many struggle to find support specifically tailored to partners.

One partner also highlighted the financial burden, particularly as a relatively young caregiver balancing work obligations alongside caregiving responsibilities, which adds further stress.

## Discussion

This qualitative study explored the experiences and needs of patients with pulmonary fibrosis in a referral center for ILD, to identify opportunities to improve palliative care. Two main themes were generated from the data: ‘See me as a person’ and ‘Guide me through the trajectory’. These findings highlight the importance of personalized, holistic care and proactive guidance throughout the illness trajectory for both patients and their partners.

The experiences described by participants in this study closely mirror findings from qualitative research in other progressive and life-limiting conditions, such as advanced cancer, heart failure, and amyotrophic lateral sclerosis. Across these disease trajectories, patients and informal caregivers consistently report uncertainty about the future, fluctuating symptom burden, emotional distress, and an increasing reliance on partners as the illness advances. The need for proactive guidance, honest communication, and reassurance that one is not navigating the illness alone has been widely documented across serious illnesses [28–31].

Patients reported varying experiences with healthcare professionals. While some felt well supported, others did not. Despite this variation, patients consistently expected empathetic and open communication. Prior research confirms that strong patient–healthcare professional relationships rely on honesty, trust, compassion, active listening, and clear, understandable language [32–34]. Such communication not only enhances satisfaction, but also strengthens coping and reduces anxiety [18,35]. These findings underscore that empathetic communication is not merely a relational skill but a core component of high-quality palliative care and requires training and courage [36]. Policy changes should prioritize systematic training in communication skills and allocate sufficient time for these interactions.

The evolving role of partners to both loved ones and informal caregivers represents a profound role change with significant emotional and practical implications. This shift often increases psychological burden and anticipatory grief, as highlighted in prior research [17]. While palliative care frameworks, such as the WHO definition [10] and the Dutch quality framework [12], recognize partners as integral to the caregiving system, evidence shows their needs remain insufficiently addressed. Structured interventions, such as tailored information, psychosocial support, and caregiver training, could be embedded into care pathways for patients with pulmonary fibrosis. Doing so could reduce stress and improve outcomes for both patients and partners [37].

Unmet informational needs emerged as a key challenge, with patients and partners expressing different priorities. This aligns with reviews on pulmonary fibrosis [17,32]. Beyond timing and format, the findings highlight the need for information that supports decision-making and coping, rather than focusing solely on disease and treatment. While healthcare professionals echo this as a challenge, addressing this requires a proactive, tailored approach and explicit coordination between patients, partners, and healthcare professionals.

Situating pulmonary fibrosis within this broader literature shows that many of the expressed experiences and needs are not disease-specific but reflect universal challenges of living with serious illness. Unlike many oncological or neurological trajectories, pulmonary fibrosis lacks clear transition points that signal when palliative care should be intensified. Together with the rarity and limited public awareness of pulmonary fibrosis, this appears to intensify patients’ uncertainty and sense of isolation compared with more widely recognized life-limiting diseases.

### Strengths and weaknesses

This qualitative study provides in-depth insights into the perspectives of patients and partners. Although a range of informal caregivers was intended, all were partners. Resulting in findings that primarily reflect partner experiences and overlooking perspectives from other roles, such as children.

Despite limited interviewing experience, a learning curve was evident, and interviewers quickly identified consistent themes. Analysis by researchers with varying levels of experience in qualitative research ensured a rigorous, nuanced interpretation.

While multiple data collection methods were used, all were analyzed together. All perspectives could be distinguished because all methods consistently aimed at capturing the perspectives of all attendees.

Dyadic interviews offered benefits, such as shared memories and dynamic interaction, encouraging deeper reflections. A potential drawback was that partners withheld openness to protect each other; this was mitigated by prior screening and ongoing checks, with one case requiring separate interviews. Generally, partners encouraged each other, and interviewers ensured balanced participation and clarified ‘we’ statements. While this format may have limited individual perspectives in sensitive areas, it provided rich relational narratives.

Although multiple focus groups were planned, most participants preferred individual interviews, resulting in only one group session, possibly reducing collective sense-making and potentially limiting the contextual richness of the findings. Consequently, findings are primarily derived from individual and dyadic interviews.

Interviews were conducted in different settings, including participants’ homes, the hospital, and online. These variations occurred due to restrictions during the COVID-19 pandemic and participants’ preferences at the time. Each setting may influence comfort levels, openness, and conversational flow, although online interviews also improved accessibility. Despite these potential differences, consistent themes emerged across all locations, suggesting that the interview setting had minimal impact on the findings.

Transferability may be limited, given the single-center design, the predominance of male participants, and the informal caregivers being partners. This may reduce the applicability of the findings to other care settings or more diverse pulmonary fibrosis populations.

We did not collect clinical staging information, as the study focused on palliative needs and communication from the time of diagnosis onward. Although staging might offer additional nuance, palliative care needs in pulmonary fibrosis often arise irrespective of disease severity. Future studies could explore potential differences in palliative needs across clinical stages.

### Implications

The findings underline the need to move beyond a medical focus and embrace a more proactive, and personalized care approach. Although healthcare professionals are increasingly trained in holistic, multidimensional care and communication, patients do not always perceive this in practice. Healthcare professionals can address this by exploring individual needs, offering tailored information about both the illness and available care options, and actively involving partners. Achieving this requires both training and a system-level support to coordinate care across disciplines and settings and maintain continuity. A personalized approach fosters trust, clarity, and a greater sense of control for both patients and caregivers.

Future research should focus on implementation strategies and the impact of personalized communication and tailored information delivery on the well-being of patients with pulmonary fibrosis. Additionally, targeted studies on information recall during diagnosis and advance care planning could provide deeper insights into the most effective ways to convey critical information to patients.

## Conclusion

The experiences of patients with pulmonary fibrosis and their partners revealed several gaps in care, summarized in two main themes that can be seen as an appeal to healthcare professionals: ‘treat me as a person’ and ‘guide me through the trajectory’.

This study shows the need for more palliative, especially more personalized palliative care to optimize the quality of life of patients with pulmonary fibrosis and their informal caregivers. Care must be tailored to the unique circumstances of patients and partners, ensuring they feel treated as a person, not as the disease. Personalized care with tailored information and a strong patient-healthcare professional relationship is essential, allowing every patient and relative to receive the support they truly need.

## Supporting information

S1 File SRQR Checklist. (DOCX)

S2 File Interview guide [38]. (DOCX)

## Abbreviations

JH: Janneke Haverkamp
LD: Lea M. Dijksman
LT: Lian Trapman
JG: Jan. C. Grutters
ST: Saskia. C.C.M. Teunissen
EdG: Everlien de Graaf
