# Supplementary material for: Experiences of palliative care for patients with pulmonary fibrosis: A qualitative exploration of patients and partners perspectives
Source: PLoS One. 2026 Oct 13;21(10):e0360649. doi: 10.1371/journal.pone.0360649 (PMC13660880; doi:10.1371/journal.pone.0360649)
Supplement: Supplementary file 2 [file pone.0360649.s002.docx]

**Supporting information 2: Interview guide**

| **Interview guide focus groups** | |
| --- | --- |
| **Focus group 1** | |
| Method: | Discussion following the river-method. (38) |
| General questions: | - What is palliative care for you? - How do you feel about the palliative care nowadays in our clinic; (what are your experiences with this?) |

| **Interview guide individual interviews** | |
| --- | --- |
| Method | River-method |
| Techniques used | - Building rapport - Summarization - Request specific examples - Employing strategic silences - Member checking during |
| General question | - Can you remember the first conversation with the pulmonologist and nurse? Can you tell me about this - Is this how a conversation usually takes place? Can you tell me about this? - Do you feel you have space in the conversation? - Does the nurse or pulmonologist talk with you about the future and end of life? - How did you feel during these conversations? - What do you think is the right time to start talking about this? - What do you expect of the healthcare professionals regarding these conversations? And from who exactly (e.g. nurse, pulmonologist, general practitioner) - Is there anything else you want to tell me about these conversations? |
